# A one-two punch against cancer: combining CDK4 inhibitors with lysosomotropic agents

**DOI:** 10.1038/s44318-025-00396-2

**Published:** 2025-02-24

**Authors:** Gerardo Ferbeyre

**Affiliations:** 1 Centre de recherche du Centre Hospitalier de l’Université de Montréal (CRCHUM), Montréal, QC H2X 0A9 Canada; 2 Département de Biochimie et Médecine Moléculaire, Université de Montréal, Montréal, QC H3C 3J7 Canada

**Keywords:** Cancer, Cell Cycle, Organelles

## Abstract

Recent work uncovers lysosomal alterations as a targetable co-vulnerability of CDK4/6 inhibitor-induced senescence in breast cancer cells.

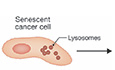

## Teaser

New work by Nehme et al (2025) demonstrates that CDK4/6 inhibitor-induced senescence in breast cancer cells leads to lysosomal alterations, rendering them vulnerable to lysosomotropic agents. These findings suggest new drug combinations to enhance cancer therapy in malignancies with senescence-associated lysosomal vulnerabilities.

In response to chemotherapy and radiation, cancer cells enter a state of proliferative arrest known as senescence. Initially, this response halts tumor progression, and through the secretion of various proinflammatory and immunomodulatory factors, senescent cells actively promote their clearance (Prasanna et al, 2021). However, not all senescent cells remain permanently dormant—some regain a proliferative capacity, often with increased aggressiveness (Milanovic et al, 2018). To prevent this resurgence, researchers have proposed that eliminating senescent cancer cells is the most effective strategy to mitigate their reactivation and potential contribution to tumor relapse (Dorr et al, 2013; Fleury et al, 2019). A variety of compounds targeting survival pathways have been repurposed to eliminate senescent cells, collectively known as senolytics. However, not all senescent cells are the same—their characteristics vary depending on the tissue of origin and the stimulus that induced senescence. As a result, there is no single universal senolytic drug capable of effectively targeting all senescent cell types.

**Figure 1:**
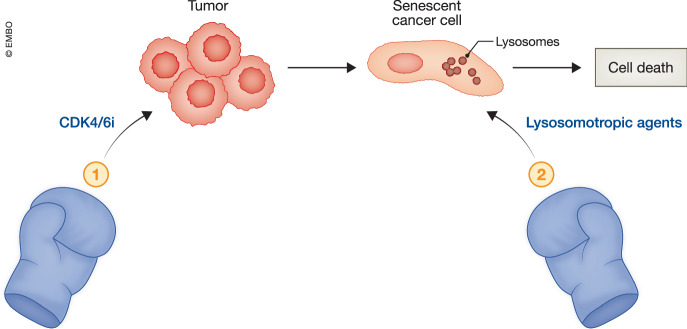
Lysosomotropic agents induce tumor cell death following CDK4/6 inhibitor-induced senescence-like arrest. One-two-punch anticancer therapies involve first inducing senescence in tumor cells, making them susceptible to a second agent that eliminates them. Here, Nehme et al (2025) introduce a novel variation of this strategy by combining the CDK4/6 inhibitor abemaciclib with the lysosomotropic agents l-leucyl-l-leucine methyl ester (LLOMe) or Salinomycin to enhance tumor cell clearance.

Among anticancer therapies that induce senescence, cyclin-dependent kinase 4 and 6 inhibitors (CDK4/6i) stand out for their ability to mimic the function of p16INK4a, one of the key regulators of senescence (Michaud et al, 2010). However, when in the current study, Nehme et al (2025) treated ER+ (estrogen receptor) or TNBC (triple-negative breast cancer*)* breast cancer cells with the CDK4/6i abemaciclib, they observed a reversible senescent-like state resistant to a variety of commonly used senolytic drugs. After analyzing the transcriptome of these senescent-like cells, Nehme et al (2025) identified altered expression of lysosomal genes, which correlated with an increase in lysosomal content, as confirmed by staining with acridine orange and other lysosomal markers. This observation led them to investigate whether these senescent cells exhibited increased sensitivity to lysosome-disrupting agents, such as l-leucyl-l-leucine methyl ester (LLOMe) and Salinomycin. Their findings revealed that sequential treatment with CDK4/6i followed by these lysosomotropic agents was highly toxic to breast cancer cells while sparing normal cells (Fig. 1). Notably, the increase in lysosomal mass rendered senescent cells vulnerable to lysosomotropic agents, even when the duration of the treatment-induced senescent-like state was brief, as observed in TNBC cell lines. This finding aligns with previous studies showing that transient senescence induced by PARP inhibitors in ovarian cancer cells was sufficient to sensitize them to the senolytic agent navitoclax (Fleury et al, 2019).

Nehme et al (2025) also investigated the mechanism underlying lysosomal alterations in breast cancer cells following CDK4/6i treatment. First, they found that in the absence of the RB tumor suppressor, CDK4/6i failed to increase lysosomal mass or enhance sensitivity to lysosomotropic agents. Second, they observed that among the tested CDK4/6 inhibitors, abemaciclib was more potent than palbociclib, a difference that correlated with abemaciclib’s ability to induce the formation of large acidic vacuoles. Finally, treatment with vacuolin-1, a compound that also increases the number of large acidic vacuoles, rendered breast cancer cells vulnerable to lysosomotropic agents, this time in an RB-independent manner.

The mechanism by which CDK4/6i-induced senescent cells undergo cell death following treatment with lysosomotropic agents remains to be elucidated. Nobel Prize laureate Christian de Duve famously referred to lysosomes as “suicide bags” due to their high content of hydrolases, which, when released, can trigger cell death. Indeed, lysosomal cathepsins have been implicated in apoptotic cell death, suggesting a potential role in this process (Foghsgaard et al, 2001). A lysosome-dependent form of necrosis was identified in worms with mutations in SRP-6, a member of the serpin family of protease inhibitors. In humans, this family comprises 37 members, categorized into nine classes (A-I) based on their structural and functional properties (Luke et al, 2007). Intriguingly, several serpins are upregulated in senescent cells (Hsieh et al, 2017), suggesting the intriguing possibility of using their inhibitors to trigger lysosomal-dependent senolysis.

Another captivating aspect of the study by Nehme et al (2025) is the mechanism underlying the tumor cell specificity of their drug combination. It remains to be determined whether normal cells resist cell death because they do not undergo lysosomal alterations or because they have protective mechanisms that neutralize lysosomal hydrolases released into the cytosol. In this regard, the authors successfully sensitized RB-negative tumor cells to lysosomotropic agents using vacuolin-1; however, this treatment was not tested in normal cells, leaving an open question about its potential effects.

In summary, the work by Nehme et al (2025) highlights lysosomes as critical therapeutic targets for the development of novel senolytic and anticancer agents, particularly in the context of hard-to-treat triple-negative breast cancer. From a clinical perspective, these findings open the door to new combination strategies that could enhance the efficacy of existing CDK4/6 inhibitors by leveraging lysosome-targeting agents. The selective toxicity of this approach toward cancer cells, while sparing normal cells, suggests a potential therapeutic window that could minimize off-target effects. Furthermore, as lysosomal dysfunction is implicated in various malignancies, this strategy could be extended beyond TNBC to other cancers that exhibit senescence-associated lysosomal vulnerabilities.
